# Distinct Roles of VEGFA and ANGPT2 in Lung Adenocarcinoma and Squamous Cell Carcinoma

**DOI:** 10.7150/jca.34693

**Published:** 2020-01-01

**Authors:** Shuang Qin, Ming Yi, Dechao Jiao, Anping Li, Kongming Wu

**Affiliations:** 1 Department of Oncology, Tongji Hospital of Tongji Medical College, Huazhong University of Science and Technology, Wuhan, 430030, China.; 2 Department of Interventional Radiology, the First Affiliated Hospital of Zhengzhou University, Zhengzhou, 450052, China.; 3 Department of Medical Oncology, The Affiliated Cancer Hospital of Zhengzhou University & Henan Cancer Hospital, Zhengzhou, 450008, China.

**Keywords:** NSCLC, VEGFA, ANGPT2, prognosis, ADC, SQC

## Abstract

**Background:** Vascular endothelial growth factor A (VEGFA) and angiopoietin 2 (ANGPT2) are key mediators in angiogenesis. The expression and clinical significance of VEGFA and ANGPT2 have been investigated in lung cancer, but the results are controversial. The specific roles of VEGFA and ANGPT2 in adenocarcinoma (ADC) and squamous cell carcinoma (SQC) are still not fully understood. To characterize it, we conducted the current study.

**Materials and Methods:** The relationships between clinic-pathological characteristics and the protein expressions of VEGFA and ANGPT2 were analyzed on tissue microarrays by immunohistochemistry (IHC) staining. Then public databases were used to evaluate the association of VEGFA and ANGPT2 mRNA expressions with clinic-pathological parameters and prognosis. Cobalt chloride (CoCl_2_) was adopted to mimic a hypoxic microenvironment and western blot was used to detect the expression of hypoxia inducible factor-1α (HIF-1α), VEGFA and ANGPT2 in lung cancer cell lines.

**Results:** IHC staining revealed that the expressions of VEGFA and ANGPT2 were enriched in lung cancer tissues compared with normal tissues. Additionally, both VEGFA and ANGPT2 protein levels were significantly associated with the tumor size and lymph node metastasis only in ADC, not SQC. More importantly, increased VEGFA and ANGPT2 protein levels were negatively correlated with overall survival (OS) of ADC individuals. Meta-analyses of 22 gene expression omnibus (GEO) databases of lung cancer implicated that patients with higher VEGFA and ANGPT2 mRNA expressions tended to have advanced stage in ADC rather than SQC. Kaplan-Meier plot analyses further verified that high levels of VEGFA and ANGPT2 mRNA were associated with poor survival only in ADC. Moreover, the combination of VEGFA and ANGPT2 could more precisely predict prognosis in ADC. In hypoxia-mimicking conditions, induced expression of HIF-1α unregulated VEGFA and ANGPT2 proteins abundance.

**Conclusion:** Our results showed hypoxia upregulated the protein levels of VEGFA and ANGPT2 in lung cancer cell lines, and the roles of VEGFA and ANGPT2 were distinct in ADC and SQC. Combined detections of VEGFA and ANGPT2 may be valuable prognostic biomarkers for ADC and double block of VEGFA and ANGPT2 may improve therapeutic outcome.

## Introduction

According to the latest cancer statistics published by American Cancer Society, lung cancer remains the leading cancer-related mortality in the United States for both male and female 1. Lung cancer is divided into two major classes based on its biology, therapy, and prognosis: small cell lung cancer (SCLC) and non-small cell lung cancer (NSCLC), the latter accounting for about 80-85% of all 2. NSCLCs consist of several subtypes, mainly including adenocarcinoma (ADC), which accounts for 50% of NSCLCs, and squamous cell carcinoma (SQC), which takes up 30% of NSCLC cases 3. In the past decade, the discoveries of driver gene mutations, such as epidermal growth factor receptor (EGFR) and kirsten rat sarcoma viral oncogene homolog (KRAS), and corresponding molecule-targeted therapies have dramatically improved the prognosis of a portion of NSCLC patients 4. But the outcome of most lung cancer patients is still far from satisfactory, which is largely due to lack of effective target, drug resistance and metastasis 5.

The hypothesis “tumor growth is angiogenesis dependent” was first proposed by Folkman in 1971 6. Tumor angiogenesis is a complex dynamic process, among which, the vascular endothelial growth factor/vascular endothelial growth factor receptor (VEGF/VEGFR) pathway 7, 8 and the angiopoietin (ANGPT)/Tie signal system 9 are the most important elements. VEGFA binds to its receptors VEGFR1 (Flt-1) and VEGFR2 (KDR/Flk-1), thus triggering multiple downstream signaling pathways, such as mitogen-activated protein kinase (MAPK) and phsphoionsitide 3-kinase (PI3Ks). Activated VEGFA signaling pathway can promote proliferation and migration of endothelial cell as well as their survival and vascular permeability 10. ANGPT1 and ANGPT2 bind with similar affinity to the extracellular domain of Tie2, an endothelial cell tyrosine kinase receptor 11. ANGPT1 is thought to drive vessel wall stabilization and maturation, mediate the migration, adhesion and survival of endothelial cell. In contrast, as an antagonistic to ANGPT1, ANGPT2, destabilizes vessel assembly, increases vessel permeability, and induces a state of vascular plasticity 12. Recently, ANGPT2 has been identified as a potent proangiogenic factor which functions in collaboration with VEGFA 13.

The clinical significance of VEGFA and ANGPT2 in lung cancer has been reported in previous studies. Although the results about the relationship among patient's clinic-pathological characteristics, prognosis and VEGFA/ANGPT2 are roughly the same, there are still some contradictions among different groups. An early meta-analysis which included 44 studies indicated the inverse relationship between VEGFA and survival in patients with NSCLC and ADC 14. However, on account of the inadequate studies on SQC (only 2), the author failed to conduct the subgroup analysis on SQC 14. Zhang et al. conducted an updated meta-analysis about the prognostic impact of VEGFA in patients with NSCLC 15. Their analyses suggested that high-VEGFA was significantly associated with poor survival in NSCLC patients and the trend was also observed in subgroup analysis of ADC and SQC patients 15. On the contrary, the results of Pajares and his colleagues indicated that high protein expressions of VEGFA and its receptors were associated with a good prognosis in patients with SQC but not in ADC 16. Apart from VEGFA, ANGPT2 is another mediator of angiogenesis. It is generally believed that ANGPT2 expression correlates with clinic-pathological features and clinical outcomes as well. Christian et al*.* observed that a higher ANGPT2 mRNA expression predicates a worse prognosis in primary breast cancer 17. A meta-analysis conducted by Xuan and his colleagues suggested that high expression of ANGPT2 in tumor tissues was significantly associated with poor survival of NSCLC, but the subgroups analysis about ADC and SQC were not performed 18. Furthermore, the levels of serum ANGPT2 were also reported to be associated with progression and prognosis in NSCLC 19. Nevertheless, most researches focused on the role of VEGFA/ANGPT2 in NSCLC while few studies centered on the distinct predictive values of VEGFA and ANGPT2 in ADC and SQC. More evidence demonstrated that ADC and SQC are fundamentally different pathological types with entirely diverse prognosis and therapeutic strategy. For example, Bevacizumab, as the first VEGFA-targeted agent, is approved only for patients with non-squamous NSCLC based on the pivotal study E4599 20. To further evaluate the expression and significance of VEGFA and ANGPT2 in ADC and SQC, respectively, tissue microarray (TMA) slides containing different pathological subtypes and large public Gene Expression Omnibus (GEO) databases were utilized. In this study, we showed that the expression of VEGFA and ANGPT2 were significantly associated with progression and clinical outcome of ADC both in mRNA and protein levels. However, the phenomenon was not observed in SQC. Our analysis strongly suggested that treatments targeting to VEGFA and ANGPT2 might be better applied to ADC.

## Materials and methods

### Human lung cancer TMA

To evaluate the protein levels of VEGFA and ANGPT2 in normal lung, ADC and SQC tissues, four commercially available human TMAs (Catalog NO. LC642, Alenabio, Xi'an, China; Catalog NO. HlugA180Su05 (two), Outdo Biobank, Shanghai, China; Catalog NO. HLugSqu150Sur01, Outdo Biobank, Shanghai, China) were purchased for IHC analysis. Specimens were obtained from patients who had undergone surgery. They must meet the following inclusion criteria: (A) histopathology confirmed lung ADC or SQC; (b) without other malignancies; (c) no systemic therapy before surgery. The clinic parameters of patients encompassed: age, gender, tumor size, TNM stage, nuclear grade, lymph metastasis, distant metastasis, survival time, and so on. LC642 contained 64 cases of SQC with age ranged from 25 to 76 years (median, 60 years). HlugA180Su05 included 94 cases of ADC and 86 matched adjacent lung tissues. There were 51 males and 43 females, and the median age was 61.5 years (range: 30-84 years). HLugSqu150Sur01 consisted of 75 pairs of primary SQC samples and corresponding adjacent lung tissues. The median age was 64 years (range: 36-78 years) with 45 patients were classified as stage I-II, while 30 patients were stage III-IV according to the 7^th^ American Joint Committee on Cancer (AJCC). Both HlugA180Su05 and HLugSqu150Sur01 have survival follow-up exceed 5 years.

### Immunohistochemical staining and quantification analysis

The specific polyclonal antibodies against VEGFA (19003-3-AP, ProteinTech, 1:200) and ANGPT2 (24613-1-AP, ProteinTech, 1:200) were utilized for IHC on TMA slides with a two-step protocol by the Biossci Biotech, Inc. 21. The VEGFA IHC image of HLugSqu150Sur01 was provided by the Shanghai Outdo Biobank. To semiquantitative evaluate VEGFA and ANGPT2 density, at least 4 fields at 200×magnification of each spot were selected and the IHC score was assessed by two individuals independently. Scoring was related to two variables: staining intensity and the percentage of positive cells. We applied Fromowitz standard to assess the intensity of staining and the percentage of positive staining tumor cells 22. The staining intensity was scored as follows: 0 (no staining), 1 (weak staining), 2 (moderate staining), 3(strong staining). The proportion of stained positive tumor cells was divided into four levels: 1 (0%-25% positive cells), 2 (26%-50% positive cells), 3 (51%-75% positive cells) and 4 (76%-100% positive cells). A score ranging from 0 to 12 was calculated by multiplying the intensity with percentage and the median score was defined as cutoff value.

### Meta-analysis for VEGFA and ANGPT2 mRNA expression on GEO databases

The method to perform the meta-analysis was described in our previous meta-analysis on SIX family 23. The electronic databases obtained from ArrayExpress were used to search for relevant GEO databases of human lung cancer with the mRNA expression of VEGFA and ANGPT2 by using the term “lung cancer”. The databases should meet the following criteria: (a) samples in the databases were human normal lung tissue or pathologically diagnosed as ADC or SQC; (b) the mRNA expression value of VEGFA and ANGPT2 were measured in the databases rather than DNA or microRNA; (c) the sample size of the database was more than 50; (d) if the same patient was included in more than one database, only the latest and most complete databases was included in the analysis; (e) the clinic-pathological and prognosis information were showed in these databases, such as grade, tumor size, lymph node metastasis, TNM stage, and clinical outcome. We adopted the median as the cutoff values of mRNA expression. The relationship between clinic-pathological parameters and VEGFA mRNA expression as well as ANGPT2 mRNA expression were assessed by the odds ratio (OR) and its corresponding 95% CI. Heterogeneity of publication bias was assessed by Cochrane Q and I^2^ test. We employed random-effect model if heterogeneity was seen between studies (I^2^> 50% or P≤ 0.05). Otherwise, we adopted fixed-effect model (I^2^≤ 50% or P> 0.05). Finally, a total of 22 independent microarray databases, were enrolled in this meta-analysis 24-45 (Table S1). The flow diagram reflecting the selection process of relevant studies was shown in Figure S1. The STATA software package (version 12.0) (Stata Corp LP, College Station, TX, USA) was employed to perform the meta-analysis.

### Analysis of public microarray

mRNA expression datasets of VEGFA, ANGPT2, and hypoxia inducible factor-lα (HIF-1α) for lung cancer were downloaded from the ArrayExpress. GSE68465, containing 443 ADC patients, and GSE4573 with 130 SQC cases were applied to evaluate the mRNA expression level in different histologic grades. GSE31210, an expression profile containing a total of 226 primary ADC patients and GSE32474, including 26 lung cancer cell lines were interrogated to assess the correlation between the mRNA expression of VEGFA, ANGPT2, and HIF-1α.

### Kaplan-Meier plotter

In this paper, we used an online analysis tool to calculate and plot Kaplan-Meier survival curves with hazard ratio (HR) and log-rank P value (http://kmplot.com/analysis/) 46. The affymetrix probe ID for VEGFA and ANGPT2 were 210513_s_at and 205572_at, respectively. The follow-up time threshold was 120 months. We used the median expression value to divide the patients into two groups, then the Kaplan-Meier survival curves were downloaded from the website and resized in Adobe Illustrator CS6.

### Cell culture and treatment

Two human lung cancer cell lines (NCI-H1299 and A549) were purchased from American Type Culture Collection (ATCC), and cultured in 1640 medium (HyClone, USA) supplemented with 10% fetal bovine serum (Gibco, USA). All cells were grown in a humidified atmosphere of 5% carbon dioxide at 37°C. CoCl_2_ was utilized to mimic a hypoxia condition, and cells were exposed to different concentration of CoCl_2_ (control, 100µM, 200µM) for 12h.

### Western blot analysis

In brief, cells were washed twice with cold phosphate buffered solution (PBS) and lysed by RIPA buffer on ice for 30 minutes and centrifuged. The cell lysates were loaded on a 10% SDS-polyacrylamide gel, and the separated proteins were then transferred onto nitrocellulose membranes. Subsequently, the membranes were incubated by the primary antibody: HIF-1α (20960-1-AP, ProteinTech, 1:1000), ANGPT2 (24613-1-AP, ProteinTech, 1:1000), VEGFA (19003-1-AP, ProteinTech, 1:1000), P21 (sc-397, Santa Cruze, 1:1000), GAPDH (10494-1-AP, ProteinTech, 1:10000) overnight at 4°C. Secondary antibody of goat anti-rabbit (1:2000) was incubated for 1h at room temperature, followed by exposure to Syngene G:BOX Chemi XT4 imaging system (Britain).

### Statistical analysis

The Student's t-test was applied to evaluate the differences between groups. A two-tailed P value <0.05 was considered statistically significant. The cumulative survival time was calculated utilizing the Kaplan-Meier method and analyzed with the log-rank test. Statistical analyses were conducted by GraphPad Prism 5.0 and SPSS 16.0. All data were presented as the mean ± standard error of mean (SEM).

## Results

### The expression of VEGFA and ANGPT2 elevated in ADC and SQC compared with normal lung tissues

**Figure 1:**
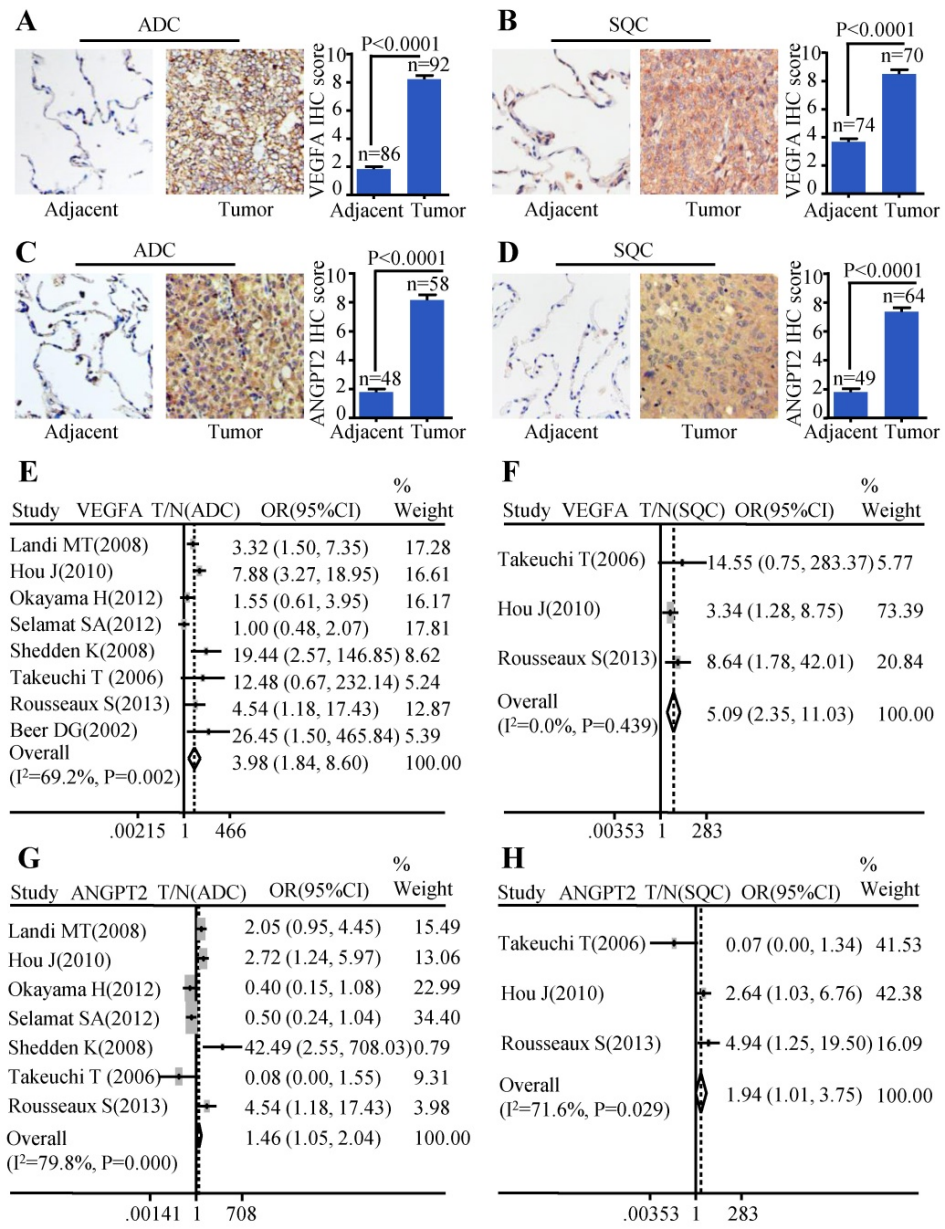
The expressions of VEGFA and ANGPT2 in ADC and SQC. Representative IHC images and scores of VEGFA in ADC vs. adjacent tissue** (A)** and SQC vs. adjacent tissue **(B)**; Representative IHC images and scores of ANGPT2 in ADC vs. adjacent tissue **(C)** and SQC vs. adjacent tissue **(D)**; The forest plot of relative mRNA expression of VEGFA between ADC and normal tissue **(E)** as well as SQC and normal tissue **(F)**; The forest plot of relative mRNA expression of ANGPT2 between ADC and normal tissue **(G)** as well as SQC and normal tissue **(H)**.

In order to evaluate the protein expression of VEGFA and ANGPT2 in ADC, SQC, and paracancerous tissues, we carried out IHC analysis on four TMAs (two HlugA180Su05, one HLugSqu150Sur01, and one LC642). The VEGFA and ANGPT2 expression with stronger brown staining particles in the cancerous tissues were mainly localized in cytoplasm and cell membrane, and with weaker cytoplasm staining in corresponding adjacent tissues. The representative images of IHC staining for noncancerous and cancers tissues were shown in Figure 1A-D. The IHC scores of tumor tissues were significantly higher than those of matched adjacent tissues (P<0.0001) (Figure 1A-D). Furthermore, we adopted a meta-analysis to evaluate whether the mRNA expression of VEGFA and ANGPT2 were consistent with the protein abundance. The patients were divided into high and low subgroups based on the median mRNA expression value. Our analysis indicated that the mRNA expression of VEGFA was increased in ADC (OR=3.98, 95% CI: 1.84-8.60, P=0.002 and I^2^=69.2%) (Figure 1E) when compared with normal lung tissues. The same tendency was seen in ANGPT2 (OR=1.46, 95% CI: 1.05-2.04, P=0.000 and I^2^=79.8%) (Figure 1G). Analysis of SQC was also proven to have the similar trend (VEGFA: OR=5.09, 95% CI: 2.35-11.03, P=0.439, I^2^=0.0%, Figure 1F; ANGPT2: OR=1.94, 95% CI: 1.01-3.75, P=0.029 and I^2^=71.6%, Figure 1H). In order to deepen our understanding about the expression of VEGFA and ANGPT2 in different histological types, namely ADC and SQC, we compared their mRNA levels between these two pathological types. The combined ORs of VEGFA were 1.73 (95% CI: 1.09-2.76; P=0.000 and I^2^=77.0%) (Figure S2A), indicating a higher VEGFA expression in ADC. Nevertheless, ANGPT2 just showed a moderate trend without reaching significance (OR=1.01, 95% CI, 0.82-1.25, P=0.651 and I^2^=0.0%) (Figure S2B).

### VEGFA expression was associated with cancer progression in ADC, not in SQC

**Figure 2:**
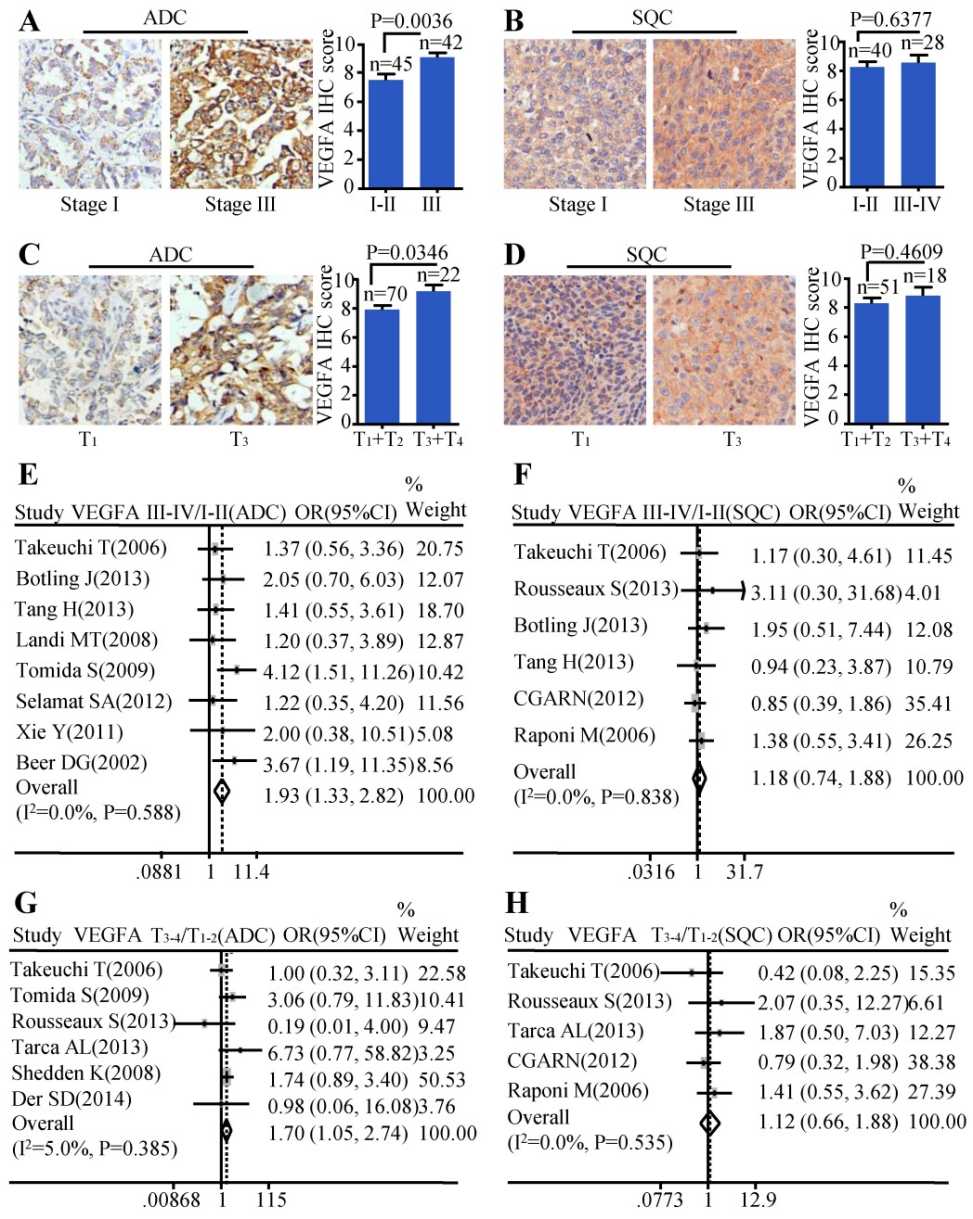
Correlation between VEGFA expression and TNM stage as well as tumor size. Representative IHC images and scores of VEGFA in different TNM stages of ADC patients **(A)** and SQC patients **(B)**; Representative IHC images and scores of VEGFA in different tumor sizes of ADC patients **(C)** and SQC patients **(D)**; The forest plot of relative mRNA expression of VEGFA at stage III-IV vs. I-II in ADC patients **(E)** as well as SQC patients **(F)**; The forest plot of relative mRNA expression of VEGFA at T_3-4_ vs. T_1-2_ in patients with ADC **(G)** and SQC **(H)**.

Moreover, we investigated the relationship between the protein level of VEGFA and clinical-pathological parameters of ADC and SQC. Patients with stage III showed stronger staining than samples with early stages (stage I-II) (P=0.0036) (Figure 2A) in ADC. The same trend was also found in ADC patients with larger tumor size. Tissues with bigger tumor size (T_3_-T_4_) had increased VEGFA expression than those with smaller tumor size (T_1_-T_2_) (P=0.0346) (Figure 2C)**.** We also analyzed the relationship between VEGFA protein level and TNM stage, tumor size in SQC. However, no statistical difference was found (Figure 2B, Figure 2D**)**. The results of meta-analysis were consistent with the protein abundance, that increased VEGFA mRNA level were significantly associated with advanced tumor stage (OR=1.93, 95% CI: 1.33-2.82, P=0.588, and I^2^=0.0%) (Figure 2E) and big tumor size (OR=1.70, 95% CI: 1.05-2.74, P=0.385, and I^2^=5.0%) (Figure 2G) in ADC patients. However, analysis in SQC showed no significance between the VEGFA mRNA expression and TNM stage (Figure 2F), tumor size (Figure 2H).

**Figure 3:**
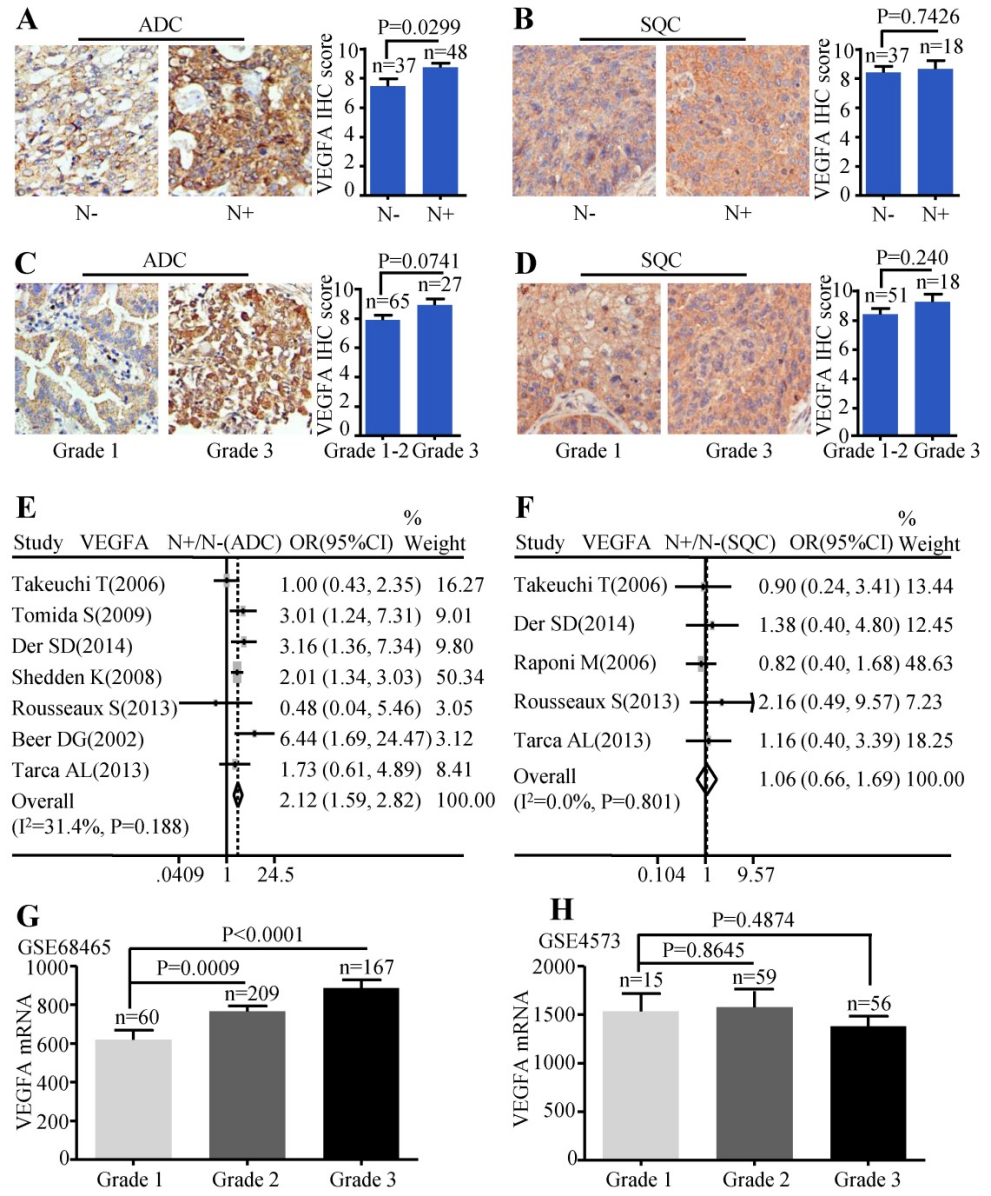
Correlation between VEGFA expression and lymph node metastasis as well as histological grade. Representative IHC images and scores of VEGFA between N- and N+ in ADC patients **(A)** and SQC patients **(B)**; Representative IHC images and scores of VEGFA in different histological grades of ADC patients **(C)** and SQC patients **(D)**; The forest plot of relative mRNA expression of VEGFA at N+ vs. N- in patients with ADC **(E)** and SQC **(F)**; Expression analysis of VEGFA in different histological grades at ADC microarray dataset GSE68465 **(G)**; Expression analysis of VEGFA in different histological grades at SQC microarray dataset GSE4573 **(H)**.

Patients with lymph node metastasis expressed more VEGFA protein than those without lymph node metastasis (P=0.0299) (Figure 3A). But, the VEGFA expression between high differentiation (grade1-2) and low differentiation (grade 3) did not reach a statistical significance (P=0.0741) (Figure 3C). Analysis conducted on SQC patients also showed no statistical difference between the protein expression of VEGFA and the different lymph node status (Figure 3B) as well as tumor grade (Figure 3D). Simultaneously, the meta-analysis suggested that increased VEGFA mRNA level was significantly associated with lymph node metastasis (OR=2.12, 95% CI: 1.59-2.82, P=0.188, and I^2^=31.4%) (Figure 3E) in ADC patients. The meta-analysis of SQC showed no significance between the VEGFA mRNA expression and N status (Figure 3F). As the data extracted were not sufficient to conduct pooled analysis for histological grade, GSE68465 including 443 ADC was interrogated to evaluate the mRNA levels of VEGFA in different grades, which showed that ADC patients with high grade expressed more VEGFA than patients with low grade (P<0.0001 and P=0.0009) (Figure 3G). GSE4573 containing a total of 130 SQC cases was employed to analyze the significance of VEGFA expression in different grades, but no statistical difference was observed (Figure 3H). Median IHC score 9 was used to divide VEGFA expression into high and low group and the correlation between VEGFA expression and clinic-pathological features of ADC patients in HlugA180Su05 was displayed in Table S2. We found the level of VEGFA protein expression was significantly related to TNM stage (P=0.041), while the correlation was not observed in other clinic-pathological characteristics including age, gender, tumor size, lymph node status and histological grade.

### ANGPT2 expression was associated with cancer progression in ADC, not in SQC

**Figure 4:**
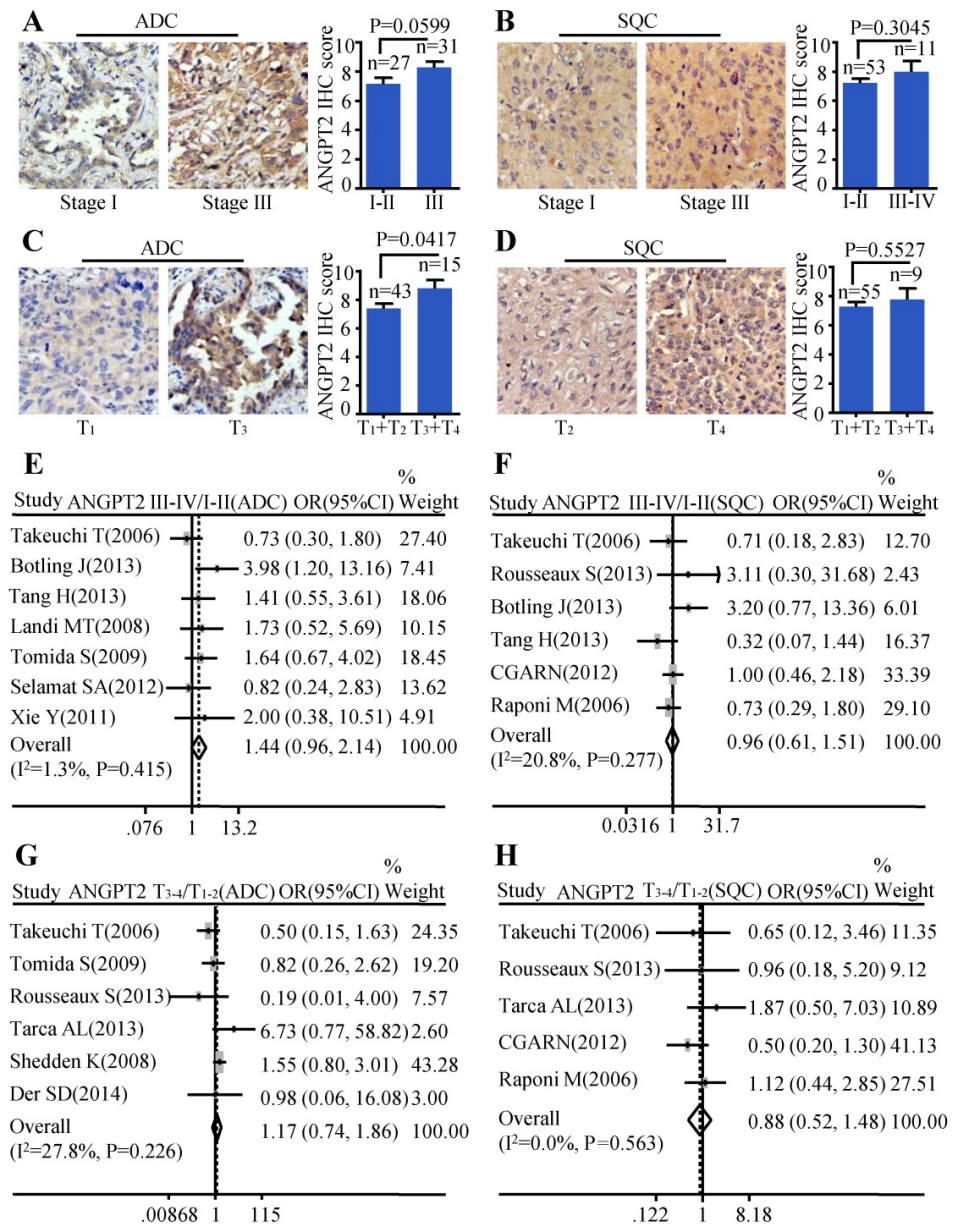
Correlation between of ANGPT2 expression and TNM stage as well as tumor size. Representative IHC images and scores of ANGT2 in different TNM stages of ADC patients **(A)** and SQC patients **(B)**; Representative IHC images and scores of ANGPT2 in different tumor sizes of ADC patients **(C)** and SQC patients **(D)**; The forest plot of relative mRNA expression of ANGPT2 at stage III-IV vs. I-II in ADC patients **(E)** as well as SQC patients **(F)**; The forest plot of relative mRNA expression of ANGPT2 at T_3-4_ vs. T_1-2_ in patients with ADC **(G)** and SQC **(H)**.

The same analysis was also conducted on ANGPT2. The relationship between ANGPT2 protein expression and TNM stage of ADC was on the verge of statistically significant (P=0.0599) (Figure 4A). However, the ANGPT2 protein abundance was higher in tumor with big size (T_3_-T_4_) than that in small size (T_1_-T_2_) (P=0.0417) (Figure 4C). Analysis performed on the relationship between protein level of ANGPT2 in SQC and clinical-pathological features mentioned above showed no statistically significance (Figure 4B, Figure 4D). The meta-analyses suggested that the correlation between ANGPT2 mRNA expression and TNM stage of ADC hovered around significance (OR=1.44, 95% CI: 0.96-2.14, P=0.415, and I^2^=1.3%) (Figure 4E) and there was no statistical difference of the ANGPT2 mRNA between the big tumor size (T_3_-T_4_) and small tumor size (T_1_-T_2_) (OR=1.17, 95% CI: 0.74-1.86, P=0.226, and I^2^=27.8%) (Figure 4G) in ADC. Statistical differences among the ANGPT2 mRNA expression and TNM stage (Figure 4F), tumor size (Figure 4H) in SQC were also not observed.

**Figure 5:**
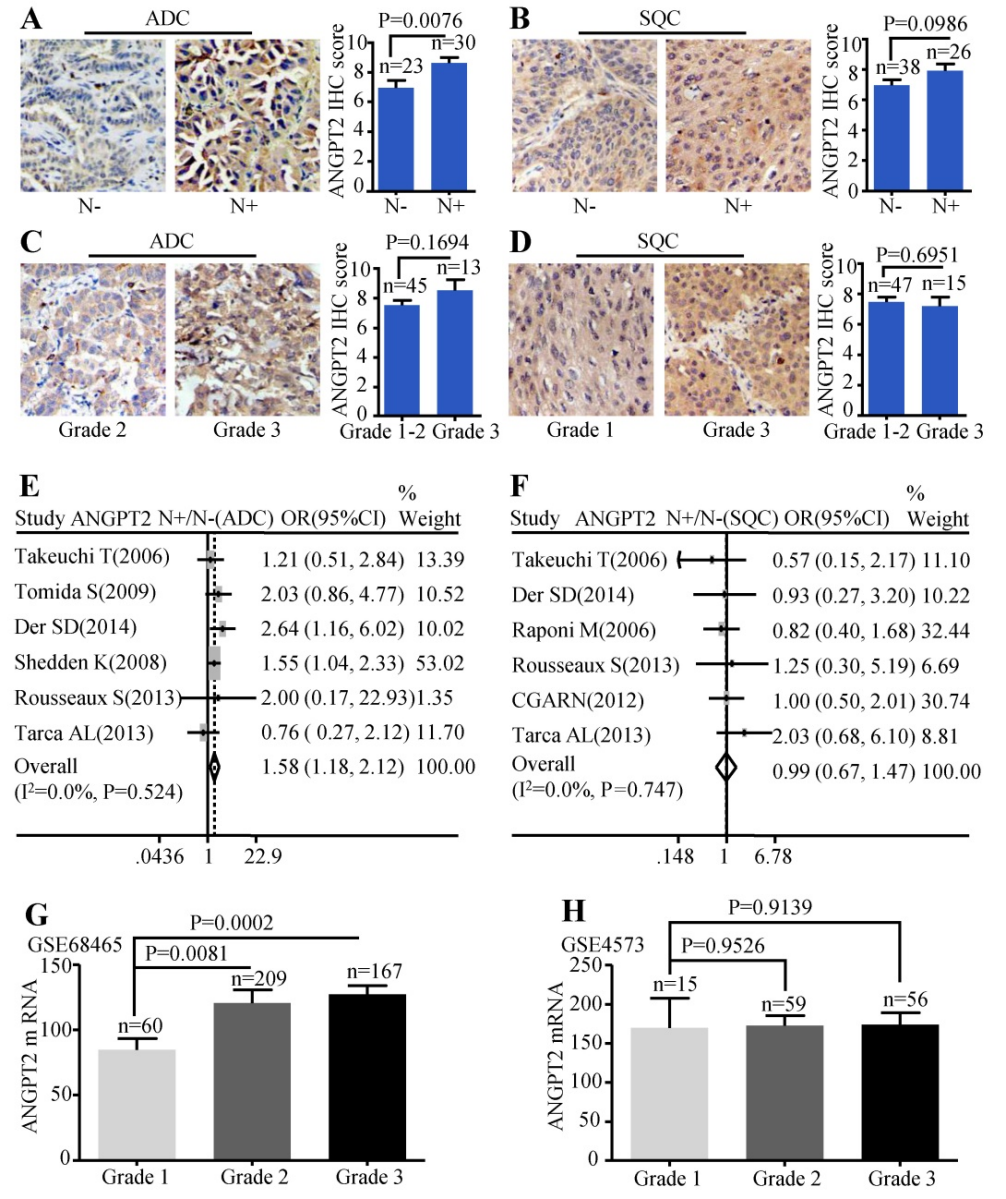
Correlation between ANGPT2 expression and lymph node metastasis as well as histological grade. Representative IHC images and scores of ANGPT2 between N- and N+ in ADC patients **(A)** and SQC patients **(B)**; Representative IHC images and scores of ANGPT2 in different histological grades of ADC patients **(C)** and SQC patients **(D)**; The forest plot of relative mRNA expression of ANGPT2 in N+ vs. N- in patients with ADC **(E)** and SQC **(F)**; Expression analysis of ANGPT2 in different histological grades at ADC microarray dataset GSE68465 **(G)**; Expression analysis of ANGPT2 in different histological grades at SQC microarray dataset GSE4573 **(H)**.

The protein level of ANGPT2 in ADC was correlated with lymph node metastasis (P=0.0076) (Figure 5A), but we failed to find significant association between tumor grade and the protein abundance of ANGPT2 (P=0.1694) (Figure 5C). Analysis conducted on SQC patients also showed no statistical difference among the protein expressions of ANGPT2 and the different lymph node status (Figure 5B), tumor grade (Figure 5D). The meta-analysis demonstrated that the mRNA level of ANGPT2 was dramatically higher in ADC patients with lymph node metastasis (OR=1.58, 95% CI: 1.18-2.12, P=0.524, and I^2^=0.0%) (Figure 5E). In contrast, there was no statistical difference between the ANGPT2 mRNA expression and lymph node status in SQC (Figure 5F). The representative dataset GSE68465 showed that the difference was statistically significant among distinct grade (P=0.0002 and P= 0.0081) (Figure 5G) in ADC while the bar graph adopted from GSE4573 certified no correlation between the ANGPT2 expression and histologic grade (Figure 5H) in SQC patients. Division of these patients into ANGPT2-high and low expression groups by median IHC score 8 revealed a strong relationship with lymph node metastasis (P=0.002) (Table S3).

### Increased expression of VEGFA and ANGPT2 predict poor survival in ADC

**Figure 6:**
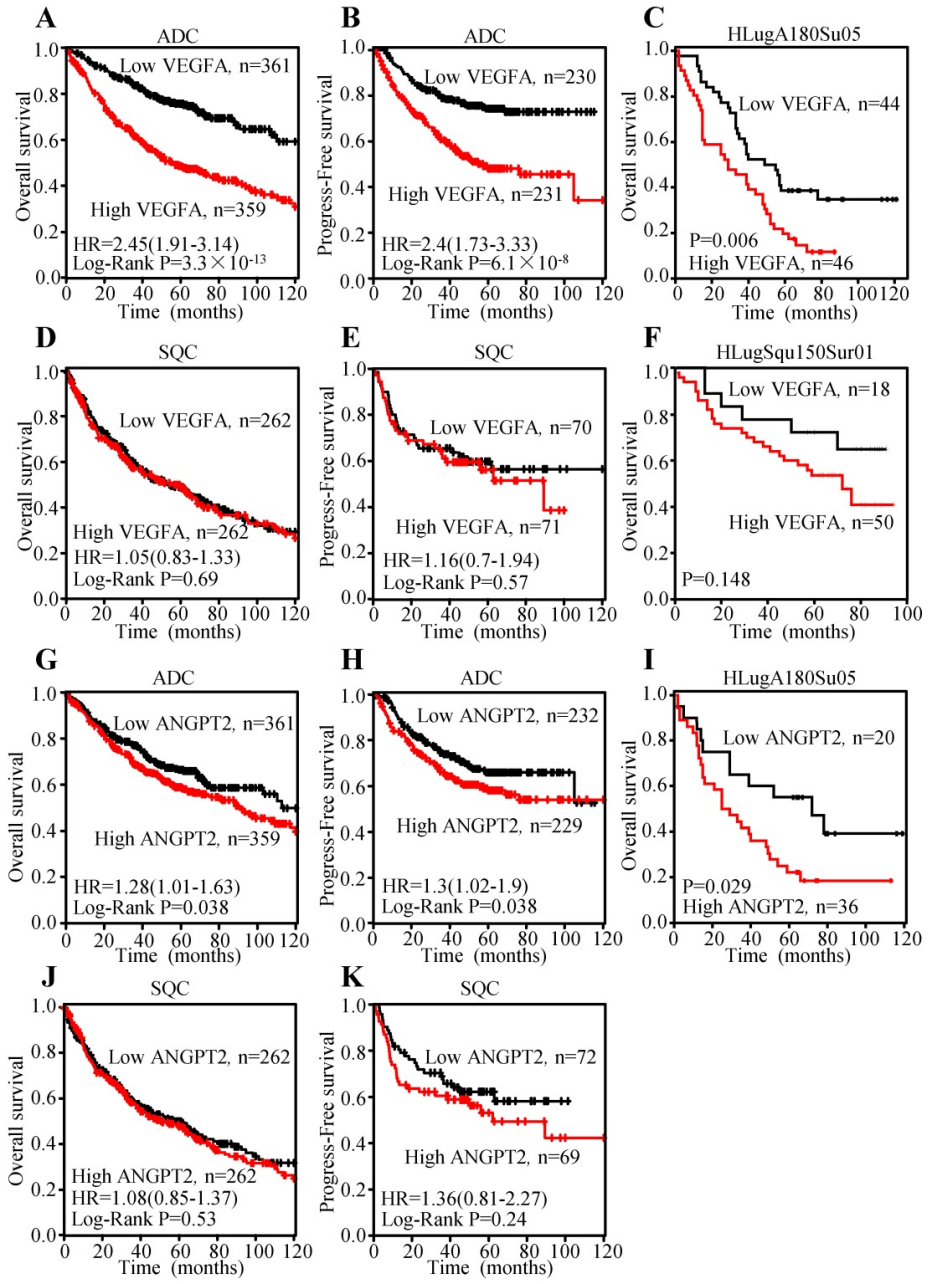
Overexpression of VEGFA and ANGPT2 predicted poor survival in patients with ADC, not SQC. Kaplan-Meier survival curves of VEGFA of ADC patients with OS **(A)** and PFS **(B)**; Kaplan-Meier survival curve of VEGFA based on the survival data from tissue microarray slide HLugA180Su05 **(C)**; Kaplan-Meier survival curves of VEGFA of SQC patients with OS **(D)** and PFS **(E)**; Kaplan-Meier survival curve of VEGFA based on the survival data from tissue microarray slide HLugSqu150Sur01 **(F)**; Kaplan-Meier survival curves of ANGPT2 of ADC patients with OS **(G)** and PFS **(H)**; Kaplan-Meier survival curve of ANGPT2 based on the survival data from tissue microarray slide HLugA180Su05 **(I)**; Kaplan-Meier survival curves of ANGPT2 of SQC patients with OS **(J)** PFS **(K)**.

To explore the prognosis value of VEGFA and ANGPT2 mRNA levels, Kaplan-Meier curves were plotted. The results indicated that patients with higher mRNA level of VEGFA had shorter overall survival (OS) (HR=2.45, 95% CI: 1.91-3.14, P<0.0001) (Figure 6A) and progression-free survival (PFS) (HR=2.4, 95% CI: 1.73-3.33, P<0.0001) (Figure 6B), which represent poor survival in ADC individuals, whilst high VEGFA expression could not serve as a predictor for OS (HR=1.05, 95% CI: 0.83-1.33, P=0.69) (Figure 6D) and PFS (HR=1.16, 95% CI: 0.7-1.94, P=0.57) (Figure 6E) in SQC patients. The analysis conducted on ANGPT2 was parallel to VEGFA, namely higher mRNA level of ANGPT2 predicted poor OS (HR=1.28, 95% CI: 1.01-1.63, P=0.038) (Figure 6G) and PFS (HR=1.3, 95% CI: 1.02-1.9, P=0.038) (Figure 6H) in ADC patients. However, ANGPT2 expression in SQC did not reach statistical significance (OS: HR=1.08, 95% CI: 0.85-1.37, P=0.53; PFS: HR=1.36, 95% CI: 0.81-2.27, P=0.24) (Figure 6J-K).

Meanwhile, we investigated the association between the protein level and prognosis. Median OS times of patients with VEGFA-low and VEGFA-high were 64.8±6.67 and 35.7±3.98 months, respectively, indicating significant difference of survival (P=0.006) (Figure 6C) in ADC individuals. The median OS times of the ANGPT2 low group was 68.3±10.56 months, while that of high group was 42.0±6.35 months (Figure 6I) in ADC patients. By contrast, analysis of VEGFA in SQC subjects did not reach to significance level (Figure 6F), which was consistent with result adopted from Kaplan-Meier plotter. Univariate Cox regression analysis was used to investigate the correlation between cumulative OS rates and clinic-pathological factors in patients with ADC. As shown in Table 1, three factors, including VEGFA expression (HR=2.139, 95% CI: 1.286-3.560, P=0.003), lymph node metastasis (HR=2.656, 95% CI: 1.546-4.565, P=0.0004), and TNM stage (HR=2.822, 95% CI: 1.681-4.735, P=0.0001) were prognostic factors for OS, whereas other clinic-pathological factors were not directly related to the clinical outcome of ADC. We performed a Forward: LR variable selection procedure using these three factors, and the VEGFA expression was identified as an independent predictive factor for the OS in ADC patients (HR=1.745, 95% CI: 1.029-2.959, P=0.039). The same univariate Cox regression analysis was conducted on ANGPT2. ANGPT2 expression, lymph node metastasis, as well as TNM stage were obviously associated with the clinical outcomes of ADC patients (Table S4).

### VEGFA expression was correlated with ANGPT2

**Figure 7:**
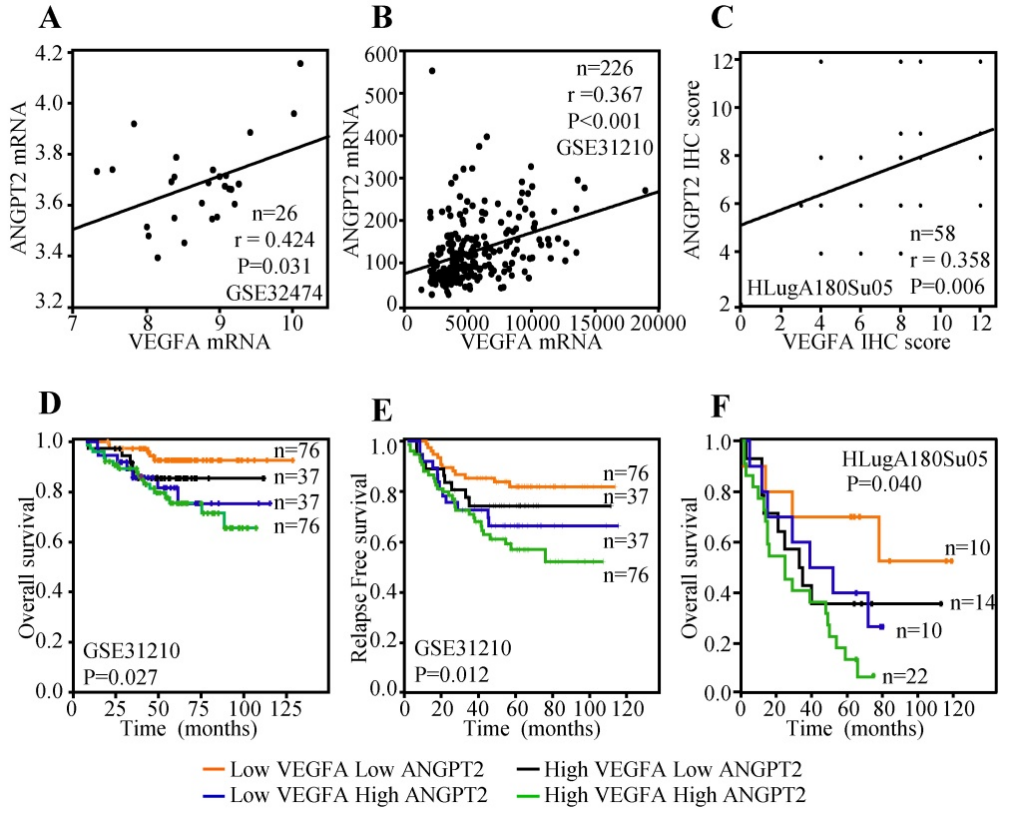
Relationship between VEGFA and ANGPT2 and combined predicted value for prognosis in patients with ADC. The correlation between VEGFA and ANGPT2 based on the mRNA level of GSE32474 cell lines **(A)** and GSE31210 ADC tissues **(B)**; The correlation between VEGFA and ANGPT2 in ADC tissues based on the protein abundance of HLugA180Su05 **(C)**; The blend Kaplan-Meier survival curves of VEGFA and ANGPT2 of ADC patients in GSE31210 with OS **(D)** and RFS **(E)**.

Previous study has indicated that the expression of VEGFA in tumor cells was positively associated with ANGPT2 and predicted poor survival 47. Herein, lung cancer cell line data reported by Kohn et al. 48, including a total of 26 lung cancer cell lines, was employed to evaluate the correlation between the mRNA expression of VEGFA and ANGPT2. The result displayed that VEGFA mRNA expression was parallel with ANGPT2 (r=0.424, P=0.031) (Figure 7A). Public dataset GSE31210, containing 226 ADC cases was also interrogated to assess the association between VEGFA and ANGPT2 at mRNA level. As expected, there was a significantly positive association between VEGFA and ANGPT2 (r=0.367, P<0.001) (Figure 7B). The IHC analysis of VEGFA and ANGPT2 for the same tissue microarray (HlugA180Su05) also showed a positive correlation between them (r=0.358, P=0.006) (Figure 7C), which was consistent with the conclusion draw from the correlation analyses of GSE32474 and GSE31210. The blend Kaplan-Meier curves in GSE31210 showed that patients with low VEGFA and low ANGPT2 at mRNA level had the longest OS and relapse free survival (RFS) time, whereas high VEGFA and high ANGPT2 predicted poorest prognosis (Figure 7D-E). The same conclusion could be acquired from the IHC score analysis of VEGFA and ANGPT2, that patients with high protein expression of VEGFA and a concomitantly high ANGPT2 expression suffered a dramatic survival reduction (P=0.040) (Figure 7F), suggesting there is a synergistic effect between VEGFA and ANGPT2.

### The association among the expressions of HIF-1α, VEGFA and ANGPT2

**Figure 8:**
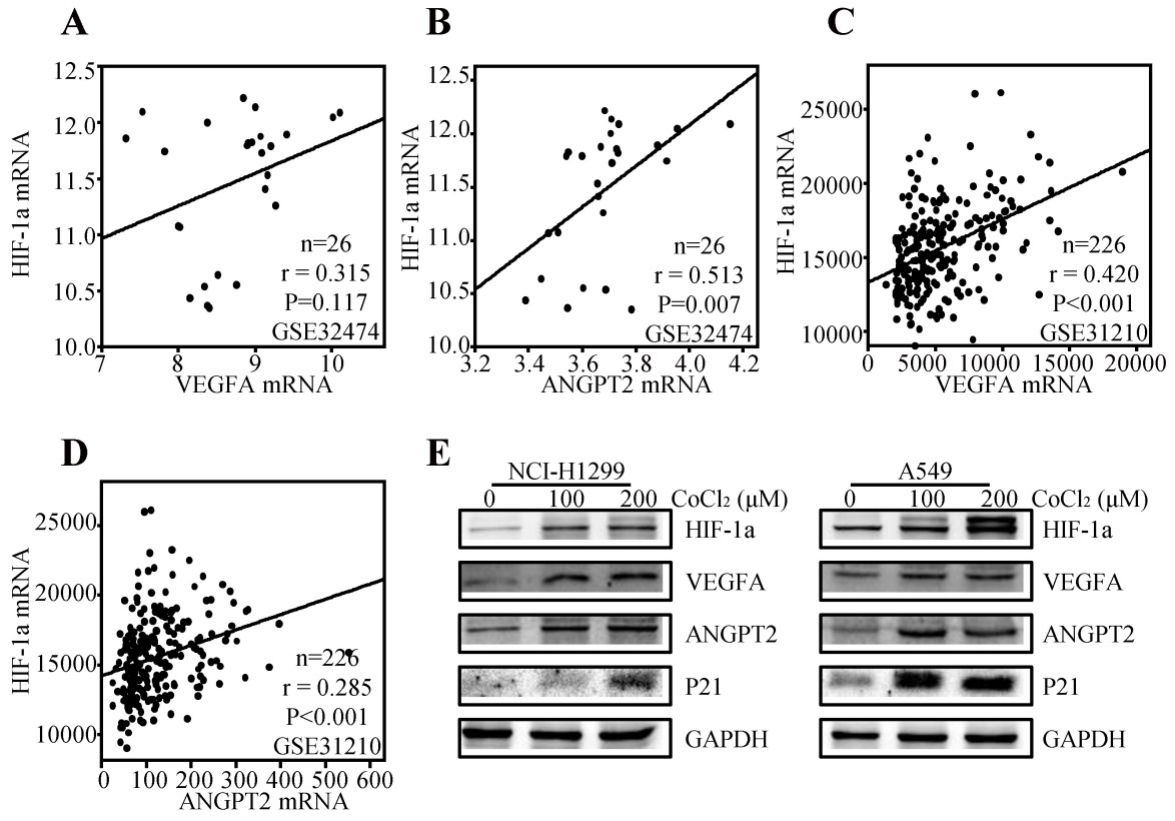
Relationship among HIF-1α, VEGFA, and ANGPT2. The correlation between HIF-1α and VEGFA based on the mRNA level of GSE32474 cell lines **(A)** and GSE31210 ADC tissues **(C)**; The correlation between HIF-1α and ANGPT2 based on the mRNA level of GSE32474 cell lines **(B)** and GSE31210 ADC tissues **(D)**; NCI-H1299 and A549 were treated with 100µM or 200µM CoCl_2_ for 12h. The antibodies used for western blot analysis was shown **(E)**.

It is well accepted that VEGFA and ANGPT2 are major angiogenesis factors, and HIF-1α is a transcription factor of VEGFA 49. Thereby, we tried to explore the regulation effect of HIF-1α on VEGFA and ANGPT2 in NSCLC. We first analyzed the correlation between the HIF-1α and VEGFA/ANGPT2 at mRNA levels. The results showed that HIF-1α mRNA expression was positively correlated with ANGPT2 both at lung cancer cell lines (r=0.513, P=0.007) (Figure 8B) and lung cancer tissues (r=0.285, P<0.001) (Figure 8D). While VEGFA was just parallel with HIF-1α in lung cancer tissues (r= 0.420, P<0.001) (Figure 8C), not in lung cancer cell lines (r=0.315, P=0.117) (Figure 8A). As HIF-1α was a major gene response to hypoxia, we used CoCl_2_ to mimic hypoxia condition 50. Treatment of NCI-H1299 and A549 cells with CoCl_2_ (100 or 200 µM) for 12h induced a significant increase in the protein level of HIF-1α, VEGFA, and ANGPT2 (Figure 8E) compared with the untreated control cells. At the same condition, hypoxia induced the protein expression of P21.

## Discussion

VEGFA is first discovered as an endothelial cell-specific mitogen and an angiogenesis inducer released by tumor cells *in vivo* and expressed in human tumors *in situ* 51. VEGFA protein has been demonstrated to be highly expressed in several NSCLC cell lines and mediated angiogenesis 52. ANGPT2, a specific extracellular ligand to Tie2, has also been showed to overexpress in NSCLC tissues in a meta-analysis 18. In our study, a total of 3388 patients in larger public GEO databases were employed to evaluate the relationship of VEGFA/ANGPT2 in ADC and SQC patients at mRNA level. Additionally, 4 tissue microarrays were used to explore the relationship between ADC/SQC and VEGFA /ANGPT2 at protein level with about 400 patients included. Up to date, we don't find other research integrating so many public databases and evaluating VEGFA/ANGPT2 in ADC and SQC at mRNA and protein levels simultaneously. Our study observed that VEGFA and ANGPT2 expressions in ADC and SQC were significantly higher compared with normal lung tissues both at mRNA and protein levels. Results in our study further indicated that VEGFA was higher in ADC compared with SQC at the mRNA level. However, there was no difference of ANGPT2 in ADC and SQC. Specifically, high VEGFA mRNA level in ADC were associated with advanced stages, large tumor size, positive lymph node metastasis, and poorly tumor cell differentiation, whilst the association was not detected in SQC. We also illustrated that high ANGPT2 was linked to lymph node metastasis both at mRNA and protein levels. However, this phenomenon was not observed in SQC. Coincidentally, a previous research reported that a significant positive association existed between ANGPT2 and lymph node metastasis in breast cancer 17.

Lung cancer represents a highly malignant and particularly aggressive cancer type, with early and widespread metastasis and poor prognosis, thus identifying a potential survival predictor is of great importance. Early in 1996, Ohta et al*.* reported that 5-year survival rates for NSCLC patients with low-VEGFA and high-VEGFA mRNA level were 77.9% and 16.7%, respectively 53. The updated meta-analysis involving 74 sets of expression of VEGFA by IHC or enzyme-linked immunosorbent assay (ELISA) in lung cancer was conducted by Zheng et al. 15. By their analysis, they concluded that the VEGFA overexpression indicated a poor prognosis in patients with NSCLC, ADC, and SQC at protein levels 15. The disparity between ours and Zheng's may arise from the different detection methods and sample size. In our study, we simultaneously investigated the VEGFA and ANGPT2 in ADC and SQC. Our results indicated that high-VEGFA and high-ANGPT2 were remarkably associated with poor prognosis of ADC, not SQC patients. Tanaka et al. also indicated that the high expression of ANGPT2 was a significant factor to predict a poor postoperative survival in NSCLC. However, they didn't perform subgroup analysis to compare its roles in ADC and SQC 47. They also demonstrated that the survival of patients with high-ANGPT2 and high-VEGFA was extremely poor, which is in accordance with our KM plotter results. According to our multivariate analysis using Cox regression, the VEGFA overexpression was found to be an independent significant prognostic factor in ADC, which was in agreement with early result reported by Imoto et al. 54. Their study indicated that VEGFA was an important prognostic factor in completely resected NSCLC, but they did not separate ADC and SQC. Similarly, they thought the VEGFA-positive rate was significantly higher in patients with ADC than in those with SQC (P=0.03). In fact, the difference in genetic changes in histologic type of lung cancer have been reported. For instance, *ras* mutation are found predominantly in ADC 55, whereas *p53* gene mutations are more frequent in SQC compared with ADC 56. The expression of angiogenic factors, which are activated from mutations such as diver gene *ras*, may be different in ADC and SQC. Those genes may control other angiogenesis factors through different pathway.

HIF-1 is a heterodimer protein complex which is composed of a constitutively expressed HIF-1β subunit and an oxygen-regulated HIF-1α subunit 57. HIF-1α is a major subunit response to hypoxia, oxidative stress and activates VEGF-induced angiogenesis 58, 59. Previous study has showed that CoCl_2_ can create a hypoxia-like state *in vitro* or *in vivo* 49. In the present study, we adopted commonly used concentration range of CoCl_2_ to create a hypoxia culture mode in two NSCLC cell lines, NCI-H1299 and A549 49, 60. Our results have confirmed that hypoxia simulated by CoCl_2_ can induce HIF-1α expression accompanying by the enhanced protein abundance of VEGFA and ANGPT2.

Indeed, anti-angiogenic therapy has been shown responses in many kinds of carcinoma 61. Bevacizumab is approved only for patients with non-squamous NSCLC due to frequently life-threatening adverse events such as pulmonary hemorrhage, particularly in patients with SQC 62. Apart from these safety concerns, patients with squamous NSCLC in several phase III trials could not benefit from the combination of antiangiogenic therapy and chemotherapy compared with chemotherapy alone 3. Our results showed that further clinical trial targeting VEGFA and ANGPT2 should exclude SQC patients based on the lack of biological impact and prognosis on SQC. ANGPT2 and VEGFA have complementary roles in regulating tumor angiogenesis and synergistic effect on survival, suggesting that dual pathway inhibition is necessary to improve treatment outcomes. A phase I study of single-agent Vanucizumab, a bispecific monoclony antibody (mAb) targeting VEGFA and ANGPT2 showed an encouraging antitumor activity and the further study is expected 63.

Our study confirms that the expressions of VEGFA and ANGPT2 in ADC and SQC are significantly higher than that in normal tissues both at mRNA and protein levels. Furthermore, the relationship between clinic-pathological parameters and expression of VEGFA and ANGPT2 supported their roles in the progression of ADC. VEGFA is positively associated with ANGPT2 in lung cancer cell lines and tumor tissues of ADC. Both VEGFA and ANGPT2 serve as poor prognostic biomarkers, and VEGFA might be an independent prognostic factor of OS in ADC patients, but not in SQC. The prognostic impact of VEGFA in ADC appears strongly associated with a concomitantly high expression of ANGPT2. Therefore, double detection of VEGFA and ANGPT2 could provide precise information for predicting the prognosis of ADC patients.

## Supplementary Material

Supplementary figures and tables.

## Figures and Tables

**Table 1:** The association among overall survival with clinic-pathological parameters and VEGFA in ADC patients

Variables	Univariate analysis		Variable selection	
	HR (95% CI)	P value	HR (95% CI)	P value
Sex (Female vs. Male)	0.752(0.457-1.238)	0.2623		
Age (>60 vs. ≤60)	1.008(0.614-1.656)	0.9733		
Tumor size (T_3_~T_4_ vs. T_1_~T_2_)	1.426(0.822-2.472)	0.2066		
Lymph node metastasis (N+ vs. N-)	2.656(1.546-4.565)	0.0004		
Grade (Grade 3 vs. Grade 1~2)	1.796(0.852-3.782)	0.1236		
Stage (III vs. I-II)	2.822(1.681-4.735)	0.0001	2.399(1.407-4.091)	0.001
VEGFA expression (High vs. Low)	2.139(1.286-3.560)	0.0034	1.745(1.029-2.959)	0.039

VEGFA, vascular endothelial growth factor A; ADC, adenocarcinoma; N-, lymph node negative; N+, lymph node positive

## Abbreviations

ADC: lung adenocarcinoma
ANGPT2: angiopoietin-2
GEO: Gene Expression Omnibus
HIF-1α: hypoxia inducible factor-lα
IHC: immunohistochemistry
NSCLC: non-small cell lung cancer
N-: lymph node negative
N+: lymph node positive
OS: overall survival
PFS: progression-free survival
SQC: squamous cell carcinoma
TMA: tissue microarray
VEGFA: vascular endothelial growth factor A
VEGFR: vascular endothelial growth factor receptor
OR: odds ratio
HR: hazard ratio
