# Supplementary material for: Distinct Roles of VEGFA and ANGPT2 in Lung Adenocarcinoma and Squamous Cell Carcinoma
Source: J Cancer. 2020 Jan 1;11(1):153–67. doi: 10.7150/jca.34693 (PMC6930396; doi:10.7150/jca.34693)
Supplement: Supplementary file 1 — Supplementary figures and tables. [file jcav11p0153s1.pdf]

Supplementary Figure 1

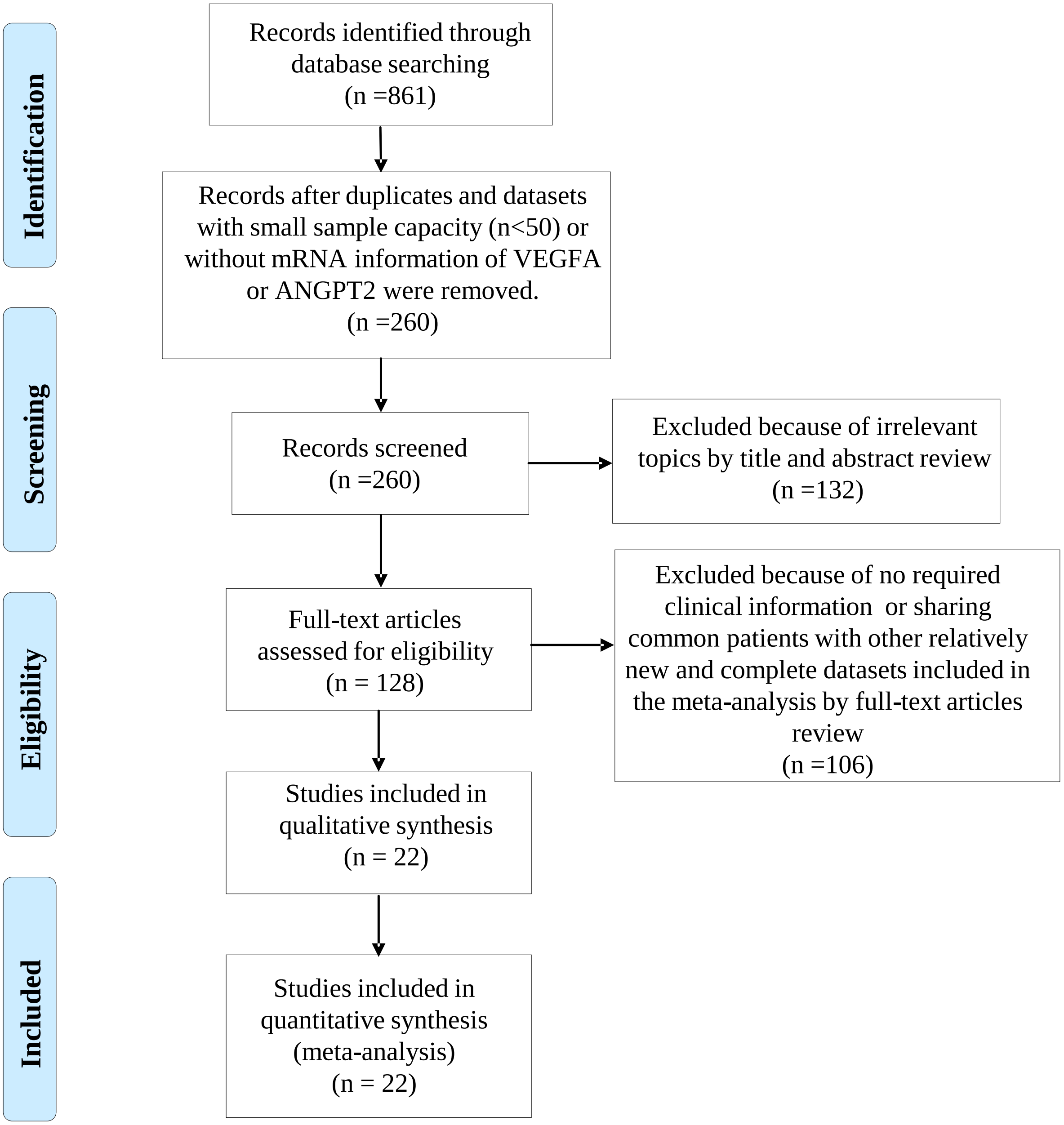

Supplementary Figure 1 Flow diagram of article selection.

Supplementary Figure 2

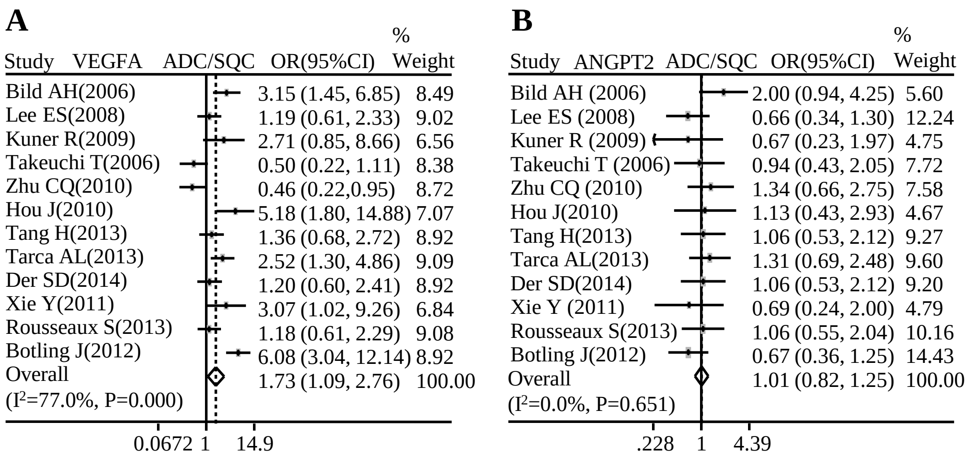

Supplementary Figure 2 Forest map of odds ratio of ADC vs. SQC. (A) The forest plot of relative mRNA expression of VEGFA between ADC and SQC patients; (B) The forest plot of relative mRNA expression of ANGPT2 between ADC and SQC patients.

Supplementary Table S1

Characteristics of the microarray databases included in the meta-analysis.

Refs.	year	Patients No.	Histology	Stage	Detection	Platform
Kuner R[24]	2009	58	NSCLC	NA	Microarray	Affymetrix Hgu133plus2.0
Zhu CQ[25]	2010	133	NSCLC	I-II	Microarray	Affymetrix Hgu133a
Xie Y[26]	2011	55	NSCLC	I-III	Microarray	Affymetrix Hgu133plus2.0
Landi MT[27]	2008	107	ADC	I-IV	Microarray	Affymetrix Hgu133a
Takeuchi T[28]	2006	163	NSCLC	I-III	Microarray	Agilent Homo sapiens 21.6K
Hou J[29]	2010	156	NSCLC	NA	Microarray	Affymetrix Hgu133plus2.0
Lu TP[30]	2010	120	NSCLC	I-IV	Microarray	Affymetrix Hgu133plus2.0
Rousseaux S[31]	2013	307	NSCLC	I-IV	Microarray	Affymetrix Hgu133plus2.0
Okayama H[32]	2012	246	ADC	I-II	Microarray	Affymetrix Hgu133plus2.0
Selamat SA[33]	2012	116	ADC	I-IV	Microarray	Illumina HumanWG-6 v3.0
Shedden K[34]	2008	443	ADC	NA	Microarray	Affymetrix Hgu133a
Beer DG[35]	2002	96	ADC	I-III	Microarray	Affymetrix HumanFull Length HuGeneFL
Bild AH[36]	2006	111	NSCLC	NA	Microarray	Affymetrix Hgu133plus2.0
Botling J[37]	2013	196	NSCLC	I-IV	Microarray	Affymetrix Hgu133plus2.0
Tang H[38]	2013	176	NSCLC	I-IV	Microarray	Illumina HumanWG-6 v3.0
Tarca AL[39]	2013	150	NSCLC	I-II	Microarray	Affymetrix Hgu133plus2.0
Der SD[40]	2014	181	NSCLC	I-II	Microarray	Affymetrix Hgu133plus2.0
Lee ES[41]	2008	138	NSCLC	NA	Microarray	Affymetrix Hgu133plus2.0
CGARN[42]	2012	133	SQC	I-IV	Microarray	Affymetrix Hgu133a
Tomida S[43]	2009	117	ADC	I-III	Microarray	Agilent-014850 G4112F
Raponi M[44]	2006	130	SQC	I-III	Microarray	Affymetrix Hgu133a
Baty F[45]	2010	56	NSCLC	I-IV	Microarray	Novachip human 34.5k

NSCLC, non-small cell lung cancer; ADC, adenocarcinoma; SQC, squamous cell lung cancer; NA, not available; CGARN, Cancer Genome Atlas Research Network.

Supplementary Table S2

Correlation between VEGFA expression and the clinic-pathological features of ADC patients in immunohistochemistry chip (HlugA180Su05).

Variables	No.	VEGFA staining		P value
		<9(low expression)	≥9(high expression)	
Age				
≤60	41	21	20	0.834 †
>60	51	25	26	
Sex				
Male	51	25	26	0.834 †
Female	41	21	20	
Stage				
I-II	45	27	18	0.041 †
III	42	16	26	
Tumor size				
T_1+T_2	70	38	32	0.143†
T_3+T_4	22	8	14	
Lymph node				
N-	37	21	16	0.234†
N+	48	21	27	
Grade				
Grade 1+2	65	35	30	0.252†
Grade 3	27	11	16	

†Pearson chi-squared test

VEGFA, vascular endothelial growth factor A; ADC, adenocarcinoma; N-, lymph node negative; N+: lymph node positive.

Supplementary Table S3

Correlation between ANGPT2 expression and the clinic-pathological features of ADC patients in immunohistochemistry chip (HlugA180Su05)

Variables	No.	ANGPT2 staining		P value
		<8(low expression)	≥8(high expression)	
Age				
≤60	26	9	17	0.639†
>60	32	13	19	
Sex				
Male	32	11	21	0.536†
Female	26	11	15	
Stage				
I-II	27	13	14	0.134†
III	31	9	22	
Tumor size				
T_1+T_2	43	18	25	0.296†
T_3+T_4	15	4	11	
Lymph node				
N-	23	13	10	0.002†
N+	30	5	25	
Grade				
Grade 1+2	45	18	27	0.780†
Grade 3	13	4	9	

†Pearson chi-squared test

ANGPT2, angiopoietin-2; ADC, adenocarcinoma; N-, lymph node negative; N+, lymph node positive.

Supplementary Table S4

The association among overall survival with clinic-pathological parameters and ANGPT2 expression in ADC patients (HlugA180Su05)

Variables	Univariate analysis		Variable selection	
	HR (95% CI)	P value	HR (95% CI)	P value
Sex (Female vs. Male)	0.814(0.428-1.547)	0.5295		
Age (>60 vs. ≤60)	0.886(0.468-1.677)	0.7089		
Tumor size (T_3~T_4 vs. T_1~T_2)	1.085(0.537-2.193)	0.8195		
Lymph node metastasis (N+ vs. N-)	3.048(1.488-6.246)	0.0023	3.048(1.488-6.246)	0.0023
Grade (Grade 3 vs. Grade 1-2)	1.337(0.519-3.445)	0.5470		
Stage (III vs. I-II)	2.523(1.313-4.851)	0.0055		
ANGPT2 expression (High vs. Low)	2.835(1.317-6.105)	0.0078		

ANGPT2, angiopoietin-2; ADC, adenocarcinoma; N-, lymph node negative; N+, lymph node positive.
